# A preoperative mortality risk assessment model for Stanford type A acute aortic dissection

**DOI:** 10.1186/s12872-020-01802-9

**Published:** 2020-12-03

**Authors:** Juntao Kuang, Jue Yang, Qiuji Wang, Changjiang Yu, Ying Li, Ruixin Fan

**Affiliations:** 1 Department of Cardiovascular Surgery, Guangdong Provincial Cardiovascular Institute, Guangdong Provincial People’s Hospital, Guangdong Academy of Medical Sciences, Guangzhou, 510080 China; 2 The Second School of Clinical Medicine, Southern Medical University, Guangzhou, 510515 China; 3 Department of Cardiovascular Surgery, Guangdong Provincial People’s Hospital, School of Medicine, South China University of Technology, Guangzhou, 510030 China

**Keywords:** Type A aortic dissections, Preoperative mortality, Scoring model

## Abstract

**Background:**

Acute aortic dissection type A is a life-threatening disease required emergency surgery during acute phase. Different clinical manifestations, laboratory tests, and imaging features of patients with acute aortic dissection type A are the risk factors of preoperative mortality. This study aims to establish a simple and effective preoperative mortality risk assessment model for patients with acute aortic dissection type A.

**Methods:**

A total of 673 Chinese patients with acute aortic dissection type A who were admitted to our hospital were retrospectively included. All patients were unable to receive surgically treatment within 3 days from the onset of disease. The patients included were divided into the survivor and deceased groups, and the endpoint event was preoperative death. Multivariable analysis was used to investigate predictors of preoperative mortality and to develop a prediction model.

**Results:**

Among the 673 patients, 527 patients survived (78.31%) and 146 patients died (21.69%). The developmental dataset had 505 patients, calibration by Hosmer Lemeshow was significant (χ^2^ = 3.260, df = 8, *P* = 0.917) and discrimination by area under ROC curve was 0.8448 (95% CI 0.8007–0.8888). The validation dataset had 168 patients, calibration was significant (χ^2^ = 5.500, df = 8, *P* = 0.703) and the area under the ROC curve was 0.8086 (95% CI 0.7291–0.8881). The following independent variables increased preoperative mortality: age (OR = 1.008, *P* = 0.510), abrupt chest pain (OR = 3.534, *P* < 0.001), lactic in arterial blood gas ≥ 3 mmol/L (OR = 3.636, *P* < 0.001), inotropic support (OR = 8.615, *P* < 0.001), electrocardiographic myocardial ischemia (OR = 3.300, *P* = 0.001), innominate artery involvement (OR = 1.625, *P* = 0.104), right common carotid artery involvement (OR = 3.487, *P* = 0.001), superior mesenteric artery involvement (OR = 2.651, *P* = 0.001), false lumen / true lumen of ascending aorta ≥ 0.75 (OR = 2.221, *P* = 0.007). Our data suggest that a simple and effective preoperative death risk assessment model has been established.

**Conclusions:**

Using a simple and effective risk assessment model can help clinicians quickly identify high-risk patients and make appropriate medical decisions.

## Background

Acute aortic dissection (AAD) is a rare life-threatening condition, and its diagnosis and treatment still remain a challenge. According to the Stanford classification, AADs involving the ascending aorta are classified as type A dissections [1]. Acute aortic dissection type A (AADA) usually requires emergency surgery in the acute phase. Without surgery, 50% of patients die within 24 h of disease onset, and the mortality rate is approximately 1–2% per hour [2]. The absence of mortality risk assessment may affect medical decision-making and resource allocation for high-risk patients and even hinder their treatment, because only timely surgical management can benefit the most patients in this group [3]. Moreover, risk stratification helps eliminate bias in high-risk patients [4]. By establishing a mortality risk assessment model, the risk of surgery can be comprehensively assessed preoperatively and the best treatment strategy can be selected, thereby reducing mortality and the incidence of complications.

Currently, several preoperative risk assessment models for cardiac surgery have been developed, such as Parsonnet model [5], European System for Cardiac Operative Risk Evaluation (Euro SCORE) [6] and Euro SCORE II [7]. These models have been widely used for assessing early preoperative mortality in various cardiac procedures. However, these scoring systems used for patients undergoing conventional cardiac surgery are not suitable for patients with AADA [8, 9]. Although there have been some risk prediction models for aorta-related surgery have been established in recent years, these models cannot be widely adopted widely because of limitations in the studies analyzing their efficacy, such as small sample sizes and inappropriate variable selection. Considering the above limitations, the predictive performance of these models is rendered questionable. Additionally, these models are based on patient samples taken from developed countries, and the models are focused on the analysis of surgical risk in patients receiving surgical treatment [3, 10–12]. A comprehensive risk assessment has rarely been performed on preoperative patients or patients awaiting surgery in the emergency department. Compared with foreign literature reports, the diagnosis and treatment of aortic dissection (AD) in China have the following characteristics: diversified clinical manifestations, younger age of onset, higher mortality during hospitalization and in the preoperative period, as well as, and more complications postoperatively [13]. Moreover, it usually takes a longer time from the onset of AD to surgical treatment for patients in developing countries. In China it takes between 2–7 days for a patient with AD onset to undergo surgical treatment, compared to only 4.5 h on average in developed countries [14]. The preoperative mortality rate of patients with AADA has not been clearly evaluated in large samples. Therefore, a preoperative mortality risk scoring system suitable for developing countries' patients is urgently required for predicting the risk of early death and for establishing emergency room guidelines in various institutions. This study aimed to establish a simple and effective risk assessment model for evaluating preoperative death risk in 673 patients with AADA within 3 days from the onset of the dissection.

## Methods

In total, 673 consecutive patients with AADA admitted to Guangdong Provincial People's Hospital between September 2017 and June 2020 were recruited, including local newly diagnosed patients in the hospital and those who were transferred to our center from other hospitals. Patients who did not undergo acute surgical treatment within 3 days from symptom onset were included. The time of onset was defined as the time when the patient first complained of severe pain in the chest or other parts, or when patients without pain complaint underwent examination and had a diagnosis of AADA. All patients were diagnosed using transthoracic echocardiography (TTE) and computed tomography angiography (CTA). Patient data, including general conditions, history, clinical manifestations, comorbidities, physical examination, laboratory tests, and imaging data, were collected (Tables 1, 2, 3). For data collection, data from our institution were given priority over from other institutions. If transferred patients were unable to undergo examinations at our institution owing to their critical condition, the data from other institutions would be collected. All data are collected and entered by specialists and reviewed by experienced and senior doctors.

**Table 1:** Demographic data, history, and presenting symptoms of patients with preoperative acute aortic dissection type A

Variable	Alive (n = 527)	Deceased (n = 146)	Total (n = 673)
DeBakey II	74 (14.04)	17 (11.64)	91 (13.52)
Age, y	53.70 ± 13.12	56.23 ± 12.39	54.25 ± 13.00
Female	92 (17.46)	31 (21.23)	123 (18.28)
Abrupt chest pain	311 (59.01)	113 (77.40)	424 (63.00)
Hypertension	310 (58.82)	81 (55.48)	391 (58.10)
Neurological disease	37 (7.02)	19 (13.01)	56 (8.32)
Marfan syndrome	30 (5.69)	7 (4.79)	37 (5.50)
Prior cardiovascular diseases	50 (9.49)	10 (6.85)	60 (8.92)
Prior cardiovascular surgery	49 (9.30)	9 (6.16)	58 (8.62)
Smoking	158 (29.98)	46 (31.51)	204 (30.31)
Anticoagulant and antiplatelet medication within the past month	42 (7.97)	5 (3.42)	47 (6.98)
Inotropic support	37 (7.02)	51 (34.93)	88 (13.08)
Ventilation	12 (2.28)	17 (11.64)	29 (4.31)

Continuous variables are reported as mean and standard deviation, and categorical variables are reported as numbers (%)

**Table 2:** Laboratory data of patients with preoperative acute aortic dissection type A

Variable	Alive (n = 527)	Deceased (n = 146)	Total (n = 673)
Lac in ABG ≥ 3 mmol/L	112 (21.25)	57 (39.04)	169 (25.11)
cTnT ≥ 200 pg/mL	38 (7.21)	21 (14.38)	59 (8.77)
WBC ≥ 15 × 10^9^/L	144 (27.32)	60 (41.10)	204 (30.31)
RBC < 3.5 × 10^12^/L	41 (7.78)	20 (13.70)	61 (9.06)
Hb < 90 g/L	13 (2.47)	11 (7.53)	24 (3.57)
ALT ≥ 200 U/L	23 (4.36)	23 (15.75)	46 (6.84)
AST ≥ 120 U/L	50 (9.49)	39 (26.71)	89 (13.22)
CREA ≥ 177 µmol/L	66 (12.52)	35 (23.97)	101 (15.01)
APTT ≥ 55 s	30 (5.69)	14 (9.59)	44 (6.54)
D-dimmer ≥ 12,000 ng/mL	206 (39.09)	95 (65.07)	301 (44.73)

Categorical variables are reported as numbers (%)

Lac, lactic acid; ABG, arterial blood gas; cTnT, Troponin T; WBC, white blood cell; RBC, red blood cell; Hb, hemoglobin; ALT, alanine aminotransferase; AST, aspartate aminotransferase; CREA, serum creatinine; APTT, activated partial thromboplastin time

**Table 3:** Imaging features of patients with acute aortic dissection type A

Variable	Alive (n = 527)	Deceased (n = 146)	Total (n = 673)
EF ≤ 50%	21 (3.98)	19 (13.01)	87 (12.93)
Average ascending aorta diameter by TTE ≥ 55 mm	54 (10.25)	33 (22.60)	87 (12.93)
Aortic insufficiency	342 (64.90)	110 (75.34)	452 (67.16)
Severe	68 (12.90)	21 (14.38)	89 (13.22)
Moderate	85 (16.13)	26 (17.81)	111 (16.49)
Minor	189 (35.86)	63 (43.15)	252 (37.44)
Moderate or massive pericardial effusion	33 (6.26)	22 (15.07)	55 (8.17)
Electrocardiographic myocardial ischemia	54 (10.25)	35 (23.97)	89 (13.22)
Sinotubular junction diameter by CTA ≥ 55 mm	22 (4.17)	16 (10.96)	38 (5.65)
Ascending aorta diameter by CTA ≥ 55 mm	84 (15.94)	28 (19.18)	112 (16.64)
FL/TL of the ascending aorta ≥ 0.75	117 (56.00)	40 (27.40)	157 (23.33)
FL/TL of the thoracic aorta ≥ 0.75	71 (13.47)	39 (26.71)	110 (16.34)
FL/TL of the abdominal aorta ≥ 0.75	40 (7.59)	25 (17.12)	65 (9.66)
Patent false lumen	207 (39.28)	93 (63.70)	300 (44.58)
RCCA involvement	76 (14.42)	41 (28.08)	117 (17.38)
IA involvement	222 (42.13)	90 (61.64)	312 (46.36)
SMA involvement	140 (26.57)	68 (46.58)	208 (30.91)
RA involvement	280 (53.13)	97 (61.64)	342 (50.82)
CIA involvement	252 (47.82)	90 (61.64)	342 (50.82)
Abdominal aortic aneurysm	23 (4.36)	6 (4.11)	29 (4.31)

Categorical variables are reported as numbers (%)

EF, ejection fraction; TTE, transthoracic echocardiography; CTA, computed tomography angiography; FL, false lumen; TL, true lumen; RCCA, right common carotid artery; IA, innominate artery; SMA, superior mesenteric artery; RA, renal artery; CIA, common iliac artery

### Statistical analysis

Patients included in the study were divided into the survivor and deceased groups. The endpoint event was preoperative death. The data were divided into a developmental dataset (505 patients) and a validation dataset (168 patients) using simple random sampling. Normally distributed continuous variables were reported as mean and standard deviation and were compared using by a two-tailed t-test. Non-normally distributed continuous variables were reported as median and quartile ranges and were compared using the Mann–Whitney U test. Categorical variables were reported as frequency and percentage and were analyzed using by χ^2^ or Fisher's exact test as needed. The stepwise multivariate analysis was performed for determining the variables that were independently associated with preoperative mortality. Odds ratios (OR) were presented with corresponding 95% confidence intervals (CI) and *P* values; *P* < 0.05 was considered statistically significant. By entering the variables with *P* < 0.05 into the logistic regression multivariate analysis, the formula was as follows: logit p = ln [p/(1 − p)] = B_0_X_0_ + B_1_X_1_ + ⋯ + B_k_X_k_. In this formula, p denotes preoperative death, and each B value was expressed as a coefficient to an independent risk factor in the final model of a particular risk.

By comparing the observed and predicted mortalities the model was then tested on the validation dataset for calibration and using the area under the receiver operating characteristic (ROC) curve for discrimination. The goodness of fit of the final model was tested using the Hosmer–Lemeshow test. In addition, tenfold cross-validation was conducted by dividing the dataset into 10 equally sized samples at random, refitting the model to each of the 10 sets comprising 90% of the data, calculating the area under the ROC curve for the unused 10% in each case and averaging over 10 areas under the ROC curves. Exclude variables with missing rates greater than 20%. Missing data are not filled in. Statistical data analysis was performed using SAS 9.4 (Cary, NC, USA).

## Results

### Univariate risk factors for death

Most of the patients are yellow, from the southern provinces of China. Among 673 patients, 146 (21.64%) patients died prior to surgery within 3 days after the onset of the disease. There were 550 men (81.72%) and 123 women (18.28%) patients, with an average age of 54.25 (± 13.00) years. The variables associated with preoperative mortality including age, abrupt chest pain, inotropic support and ventilation (Table 4). Other statistically significant univariate risk factors included lactic acid in arterial blood gas (Lac in ABG) ≥ 3 mmol/L, Troponin T (cTnT) ≥ 200 pg/mL, white blood cell count (WBC) ≥ 15 × 10^9^/L, D-dimmer ≥ 12,000 ng/mL, ejection fraction (EF) ≤ 50%, moderate or massive pericardial effusion, electrocardiographic myocardial ischemia, CTA measured sinotubular junction diameter of ≥ 55 mm, patent false lumen, and right common carotid artery (RCCA) involvement. (Tables 2, 3). No statistically significant difference was observed between the survivor and deceased groups [84 (15.94%) vs. 28 (19.18%), *P* = 0.299] in the CTA measured ascending aorta diameter of ≥ 55 mm. However, in the average ascending aorta diameter measured using TTE, there was a statistically significant difference between the groups [54 (10.25%) vs. 33 (22.60%), *P* < 0.001]. Preoperative death was strongly associated with false lumen (FL)/true lumen (TL) ratio of ≥ 75% of the ascending aorta, thoracic aorta, and abdominal aorta. Complications from AD had a significant impact on preoperative mortality.

**Table 4:** Variables associated with mortality (n = 505)

Variable	OR	95% CI	*P* value
Age	1.015	1.001–1.030	0.038
Abrupt chest pain	2.378	1.555–3.637	< 0.001
Inotropic support	7.108	4.412–11.452	< 0.001
Ventilation	5.654	2.635–12.136	< 0.001
Lac in ABG ≥ 3 mmol/L	2.404	1.616–3.576	< 0.001
cTnT ≥ 200 pg/mL	2.292	1.293–4.065	0.005
WBC ≥ 15 × 10^9^/L	1.938	1.317–2.850	0.001
RBC < 3.5 × 10^12^/L	1.931	1.091–3.417	0.024
Hb < 90 g/L	3.299	1.445–7.536	0.005
ALT ≥ 200 U/L	4.188	2.271–7.724	< 0.001
AST ≥ 120 U/L	3.801	2.368–6.103	< 0.001
CREA ≥ 177 µmol/L	2.447	1.536–3.898	< 0.001
D-dimmer ≥ 12,000 ng/mL	3.363	2.239–5.052	< 0.001
EF ≤ 50%	3.748	1.953–7.195	< 0.001
Average ascending aorta diameter by TTE ≥ 55 mm	2.650	1.635–4.294	< 0.001
Aortic insufficiency			
Severe	1.843	0.988–3.438	0.055
Moderate	1.825	1.017–3.277	0.044
Minor	1.989	1.230–3.216	0.005
Moderate or massive pericardial effusion	2.656	1.496–4.716	0.001
Electrocardiographic myocardial ischemia	2.789	1.737–4.478	< 0.001
CTA measured sinotubular junction diameter of ≥ 55 mm	2.892	1.475–5.669	0.002
FL/TL of the ascending aorta ≥ 0.75	2.269	1.529–3.369	< 0.001
FL/TL of the thoracic aorta ≥ 0.75	2.418	1.548–3.777	< 0.001
FL/TL of the abdominal aorta ≥ 0.75	2.592	1.511–4.447	0.001
Patent false lumen	2.930	1.983–4.327	< 0.001
RCCA involvement	2.395	1.547–3.709	< 0.001
IA involvement	2.369	1.612–3.481	< 0.001
SMA involvement	2.528	1.724–3.708	< 0.001
RA involvement	1.890	1.272–2.807	0.002
CIA involvement	1.877	1.278–2.756	0.001

CI, confidence interval; OR, odds ratio; Lac, lactic acid; ABG, arterial blood gas; cTnT, Troponin T; WBC, white blood cell; RBC, red blood cell; Hb, hemoglobin; ALT, alanine aminotransferase; AST, aspartate aminotransferase; CREA, serum creatinine; EF, ejection fraction; TTE, transthoracic echocardiography; CTA, computed tomography angiography; FL, false lumen; TL, true lumen; RCCA, right common carotid artery; IA, innominate artery; SMA, superior mesenteric artery; RA, renal artery; CIA, common iliac artery

### Preoperative prediction model

**Fig. 1:**
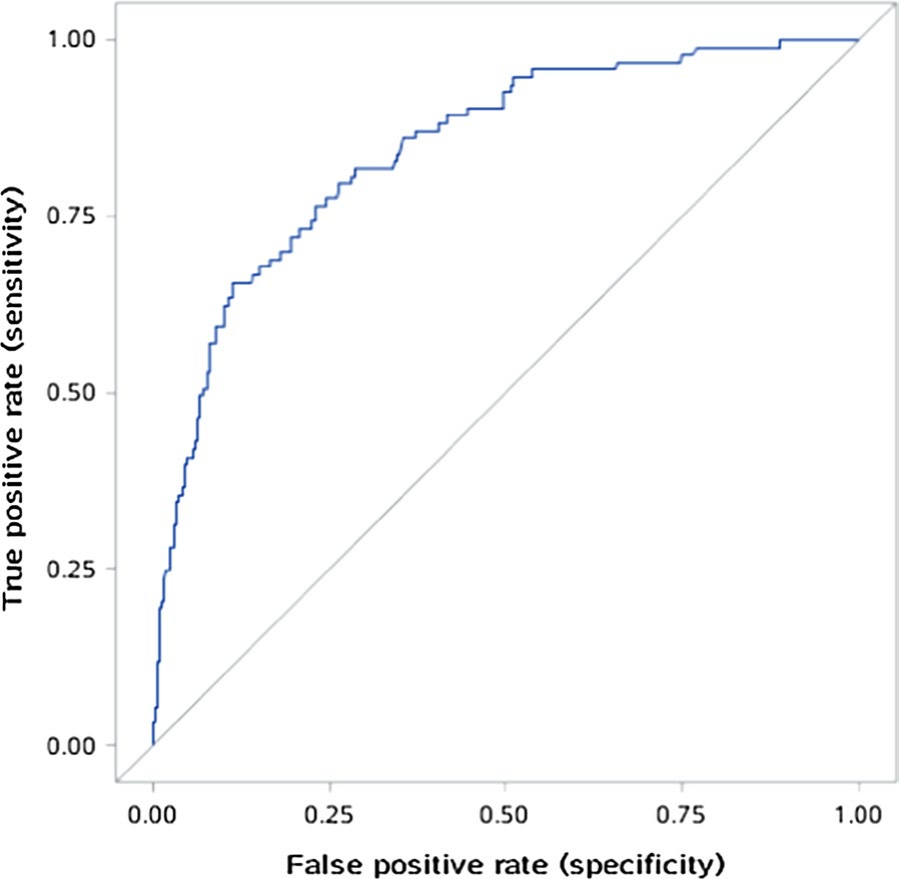
Receiver operating characteristic curve (blue line) for the developmental dataset (n = 505)

**Fig. 2:**
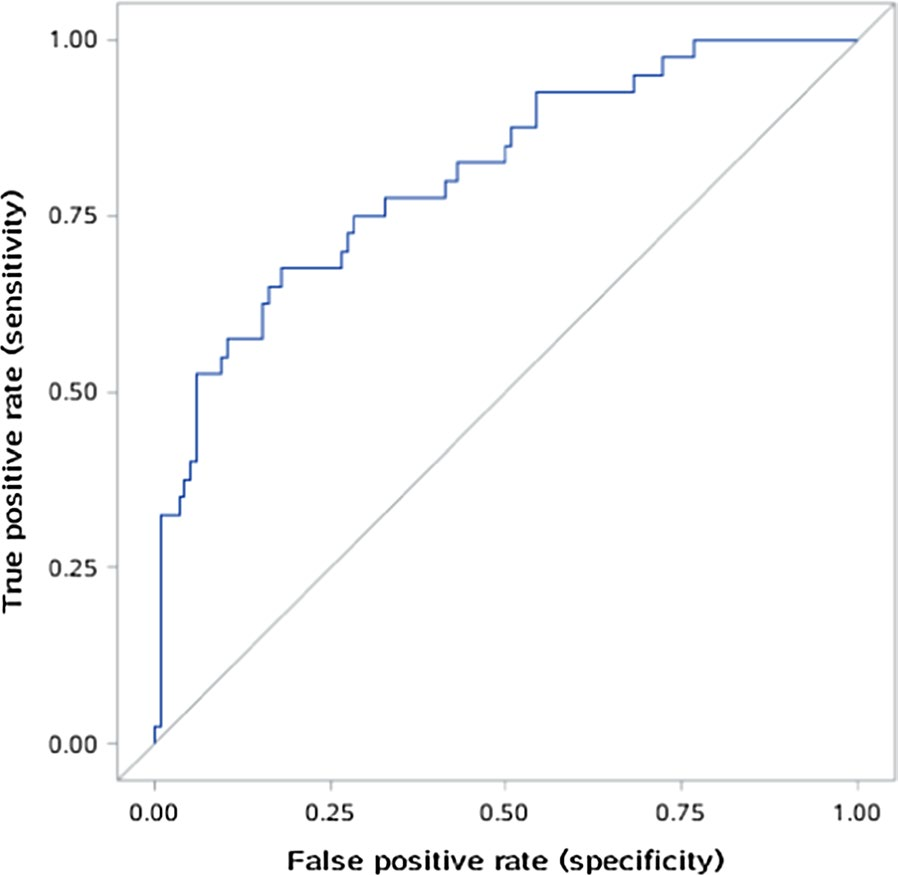
Receiver operating characteristic curve (blue line) for the validation dataset (n = 168)

In the multivariate logistic regression analysis, the following independent variables increased preoperative mortality: age (OR = 1.008, *P* = 0.510), abrupt chest pain (OR = 3.534, *P* < 0.001), Lac in ABG ≥ 3 mmol/L (OR = 3.636, *P* < 0.001), inotropic support (OR = 8.615, *P* < 0.001), electrocardiographic myocardial ischemia (OR = 3.300, *P* = 0.001), innominate artery (IA) involvement (OR = 1.625, *P* = 0.104), RCCA involvement (OR = 3.487, *P* = 0.001), superior mesenteric artery (SMA) involvement (OR = 2.651, *P* = 0.001), FL / TL of the ascending aorta of ≥ 0.75 (OR = 2.221, *P* = 0.007) (Table 5). Based on the results of the multiple regression analysis, a risk scoring model was established. The formula for preoperative death risk score is as follows: log p = ln [p/(1 − p)] = − 3.83 + 0.008 × (age) + 1.262 × (abrupt chest pain) + 1.291 × (Lac in ABG) + 2.154 × (inotropic support) + 1.194 × (electrocardiographic myocardial ischemia) + 0.486 × (IA involvement) + 1.249 × (RCCA involvement) + 0.975 × (SMA involvement) + 0.798 × (FL/TL of the ascending aorta of ≥ 0.75). The Hosmer–Lemeshow goodness of fit for the logistic regression model was significant (χ^2^ = 3.260, df = 8, *P* = 0.917), and the area under the ROC curve was 0.8448 (95% CI 0.8007–0.8888). Calibration of the validation dataset was significant (χ^2^ = 5.500, df = 8, *P* = 0.703), and the area under the ROC curve was 0.8086 (95% CI 0.7291–0.8881) (Figs. 1 and 2); both the developmental and validation models retained very good discrimination. The resulting average areas of tenfold cross-validation was 0.8057 (range, 0.6986–0.8772), which was very similar to that of the validation set in our initial analysis.

**Table 5:** Preoperative prediction model

Variable	OR	95% CI	Coefficient & score assigned	*P* value
Age	1.008	0.985–1.030	0.008	0.510
Abrupt chest pain	3.534	1.872–6.672	1.262	< 0.001
Lac in ABG ≥ 3 mmol/L	3.636	2.047–6.461	1.291	< 0.001
Inotropic support	8.615	4.361–17.019	2.154	< 0.001
Electrocardiographic myocardial ischemia	3.300	1.621–6.718	1.194	0.001
IA involvement	1.625	0.905–2.918	0.486	0.104
RCCA involvement	3.487	1.706–7.127	1.249	0.001
SMA involvement	2.651	1.527–4.602	0.975	0.001
FL/TL of the ascending aorta of ≥ 0.75	2.221	1.238–3.984	0.798	0.007

CI, confidence interval; OR, odds ratio; ABG, arterial blood gas; FL, false lumen; TL, true lumen; RCCA, right common carotid artery; IA, innominate artery; SMA, superior mesenteric artery

## Discussion

In our study, clinical manifestations, laboratory tests, and imaging features were significantly associated with AADA preoperative deaths. Additionally, a simple and quick bedside scoring model was established which surgeons can quickly use to perform preoperative risk assessments, arrange for the operation, and establish faster channels for effectively treating patients with AADA, especially those deemed to be at high risk for preoperative death. For patients with poor prognoses, model predictions are not necessarily used to deny aggressive treatment.

In agreement with the general cardiac surgery findings [15, 16], ALT ≥ 200 U/L and AST ≥ 120 U/L were associated with preoperative death (*P* < 0.001) in our study. Serum albumin level, a widely used evaluation indicator of liver dysfunction, was not measured in most patients in our study, and therefore, was not included as a variable.

In our study, age was a predictive factor, which concurred with the findings of previous studies [11, 17, 18]. Patients' age ranged from 9–91 years, and only 3 patients were < 20 years old; thus, the model prediction results for young patients might not be accurate. This observation may relate to the longer average time for patients with AADA to undergo surgical treatment in China [14].

The model included variables with electrocardiographic myocardial ischemia. Electrocardiography is very important for the early differential diagnosis of patients with acute severe chest pain owing to the simplicity and convenience of performing this test. AD and coronary heart disease (CHD) are both high-risk cardiovascular diseases, and the case of acute myocardial ischemia (AMI) with AD is a clinically rare critical illness, with a reported incidence of approximately 1–5% [19, 20]. It is generally believed that AD can cause AMI. The mechanism involves the intima of the AD extending to the coronary openings, or the false lumen compressing the coronary artery, resulting in myocardial ischemia and hypoxia, leading to AMI [21, 22]. The variable CHD was not included in our study, because only a small proportion of patients were diagnosed using coronary angiography or other objective methods; moreover, the definition of CHD and its application in retrospectively collected data may also affect the results in terms of prevalence. Furthermore, in our study, a cTnT level of ≥ 200 pg/mL was associated with preoperative death (*P* = 0.005). Considering that most patients with angina pectoris had more severe vascular disease, coronary artery stenosis and thrombosis caused greater myocardial and vascular damage, which increased cTnT levels.

In our study, the FL/TL ratio of the ascending aorta, thoracic aorta, and abdominal aorta were all ≥ 0.75 and were statistically significant in the univariate analysis; however, only the FL/TL ratio of the ascending aorta of ≥ 0.75 was selected among the results of the logistic regression multivariate analysis. Vessel malperfusion could lead to severe complications [12]. The ascending aorta connects the three branches of the aortic arch on its upward course and connects the aortic root on its downward course; the abdominal aorta consists of important abdominal blood vessels, while the thoracic aorta has none. Recently, wall shear stress has been used for evaluating each section of the aorta, and it was found that the aortic arch was in the position prone to rupture in patients with AADA [23, 24]. We speculated that the ascending aorta malperfusion might be a high-risk factor associated with the extension of rupture. Further study is needed to support this finding.

Ventilation, inotropic support, cardiopulmonary resuscitation, and preoperative syncope are related to in-hospital deaths, indicating that patients are in critical condition [11]. In our study, only inotropic support was selected in the final model. We suspected that because the number of patients who underwent these management methods was small and could not be included in the model. Since most patients were referrals from other hospitals, some patients might not have been able to transfer owing to their critical condition; these patients were likely to account for under estimation of the actual incidence.

With each additional malperfused organ system, the risk of mortality and complications increases progressively [25]. Previous studies have shown that coronary artery involvement is an independent risk factor for serious postoperative complications or death, whereas the involvement of the supra-aortic artery or celiac vessels (celiac trunk or SMA) is not a risk factor for mortality or complication rates [26, 27]; however, contradictory results were noted in our study (SMA involvement [OR = 2.651; 95% CI 1.527–4.602]). Previous studies have reported malperfusion syndrome in 26.7% of the 221 AADA patients, involving a single organ system in 64.4%, two systems in 27.1%, three in 5.1%, and four in 3.4% [28, 29]. In our study, a lower but similar distribution of the involvement of important branches of the aorta (RCCA, IA, SMA, RA, CIA) was observed: one in 17.7%, two in 26.0%, three in 22.1%, four in 12.5%, and five in 12.5%. Impaired consciousness was most likely caused by acute occlusion of supra-aortic branches, such as RCCA or IA, and unlike chest pain, abdominal pain caused by ischemia of the celiac trunk artery or SMA was often atypical or was masked by chest pain. Morbidity and mortality increased rapidly if supra-aortic vessels were involved or if visceral organs were ischemic. Our findings have been supported by the findings of Di Eusanio M [30].

Owing to the uneven distribution of medical resources in China, many regional hospitals cannot perform surgical treatment of AD independently; they can only refer patients to higher-level hospitals or request experts at higher-level hospitals for assistance with consultation and surgical treatment. Thus, most patients with AADA admitted in our hospital were transferred from other hospitals. These patients underwent a preliminary examination at the regional hospital since the onset of the disease and were considered to be diagnosed with A; they were then referred to our hospital for treatment, where they underwent relevant examinations and preparations prior to surgery. This process took an average of 3 days; therefore, the inclusion criteria for being unable to undergo surgical treatment within 3 days from the onset of disease was included. According to previous reports, the mortality rate of patients with AADA who did not undergo surgical treatment had increased dramatically over time, with an hourly mortality rate of 1% and a 90-day expected mortality rate of 70–90% [10, 31].

### Limitations

This is a single-center retrospective study and hence, the results are susceptible to a selection bias. We used an endpoint time of preoperative death within 3 days, which was the sole outcome. However, this limitation does not mitigate the importance of other outcome variables, such as nonfatal adverse events, complications, and patient functional status. Further study is required for analyzing these outcomes.

## Conclusions

In our study, the individual risk factors for preoperative mortality were analyzed in patients with AADA, and a simple and effective risk assessment method was established to help clinicians quickly identify high-risk patients and make appropriate medical decisions. Further multicenter studies are needed for verifying the prospective data and the results of our study.

## Data Availability

The datasets used and/or analysed during the current study available from the corresponding author on reasonable request.

## Abbreviations

AAD: Acute aortic dissection
AADA: Acute aortic dissection type A
Euro SCORE: European system for cardiac operative risk evaluation
AD: Aortic dissection
TTE: Transthoracic echocardiography
CTA: Computed tomography angiography
Lac: Lactic acid
ABG: Arterial blood gas
cTnT: Troponin T
WBC: White blood cell
RBC: Red blood cell
Hb: Hemoglobin
ALT: Alanine aminotransferase
AST: Aspartate aminotransferase
CREA: Serum creatinine
APTT: Activated partial thromboplastin time
EF: Ejection fraction
FL: False lumen
TL: True lumen
RCCA: Right common carotid artery
IA: Innominate artery
SMA: Superior mesenteric artery
RA: Renal artery
CIA: Common iliac artery;
OR: Odds ratios
CI: Confidence intervals
ROC: Receiver operating characteristic
CHD: Coronary heart disease
AMI: Acute myocardial ischemia

## Declarations

This retrospective study was approved by the ethics committee of Guangdong Provincial People's Hospital, Guangdong Academy of Medical Sciences, Guangzhou, China. Written informed consent was obtained from the patients and their legal relatives.
